# Protective effects of AER-271 in acute-phase radiation-induced brain injury in rats: reduction of brain edema, inflammation, apoptosis and maintenance of blood-brain barrier integrity

**DOI:** 10.3389/fphar.2025.1534729

**Published:** 2025-05-08

**Authors:** Yaozu Xiong, Yifei Wang, Mingyue Li, Changhua Yu, Yusuo Tong, Xiaoting Xu

**Affiliations:** ^1^ Department of Radiotherapy for Oncology, The First Affiliated Hospital of Soochow University, Suzhou, China; ^2^ Department of Pathology, Children’s Hospital of Soochow University, Suzhou, China; ^3^ Department of Radiotherapy, Second Hospital of Radiation, Soochow University, Suzhou, China; ^4^ Department of Tumor Radiotherapy, Huai’an First Hospital, Nanjing Medical University, Huai’an, China

**Keywords:** AQP4, AER-271, radiation-induced brain injury, cerebral edema, inflammation, blood-brain barrier, Apoptosis

## Abstract

**Objective:**

The expression changes of aquaporin-4 (AQP4) in radiation-induced brain jinjury (RIBI) and whether it is involved in the pathologic development of RIBI are currently unknown. In this study, we constructed a RIBI model by whole-brain radiation of Sprague-Dawley (SD) rats and tried to reveal the role of AQP4 in RIBI. The specific inhibitor AER-271 was used to inhibit the expression of AQP4 in RIBI to explore its neuro-protective effect.

**Methods:**

SD rats were randomly divided into Sham group and IR group. The trend and role of AQP4 in RIBI were explored by H&E staining, Western blot, brain tissue water content measurement, Evans blue (EB) osmolality assay, and immunofluorescence staining. Then SD rats were randomly divided into Sham group, AER-271 group, IR group and IR+AER-271 group to investigate the neuroprotective effects of AER-271 by H&E staining, Western blot, brain tissue water content measurement, EB osmolality assay, immunofluorescence staining, qRT-PCR and Elisa.

**Results:**

Radiation promoted the expression of AQP4 in rat brain tissue, leading to its “depolarized” distribution. The expression level of AQP4 correlated with the severity of cerebral edema. Treatment with AER-271 reduced cerebral edema, attenuated inflammation and apoptosis, and maintained the integrity of the blood-brain barrier (BBB) in RIBI rats.

**Conclusion:**

AQP4 is involved in regulating the subsequent inflammatory response, BBB injury and apoptosis by mediating the development of cerebral edema during the acute phase of RIBI. AER-271 is expected to be a promising therapeutic candidate for the treatment of RIBI by inhibiting the expression of AQP4.

## 1 Introduction

Radiotherapy is an important treatment for primary brain tumors (such as glioblastoma) and metastatic brain tumors. Its application alone or combined with other treatment measures, such as radiotherapy combined with tumor treating fields (TT fields) therapy, is of great help to improve the prognosis of brain tumors (Shah et al., 2025). However, even though radiotherapy techniques have been substantially improved today, the occurrence of RIBI is still unavoidable (Zhang et al., 2023). From the primary brain injury caused by ionizing radiation to the subsequent series of secondary injuries, RIBI involves complex pathological mechanisms and cascading responses (Yang et al., 2017). According to the time of onset, RIBI can be divided into acute type, early delayed type and late delayed type. Acute RIBI occurs within 1–2 weeks after radiation and is characterized by cerebral edema, neuro-inflammation, BBB destruction, and apoptosis. If these injuries persist, they will cause neuronal cell death and lead to cognitive dysfunction in the late stage (Yang et al., 2017; Balentova and Adamkov, 2015). Therefore, it is important to reduce the pathological damage of RIBI in the acute phase to improve the prognosis.

In the central nervous system (CNS), a variety of cells including: neuronal cells, astrocytes, microglia, vascular endothelial cells, and oligodendrocytes have been implicated in the pathogenesis of RIBI (Hwang et al., 2006). Astrocytes are known as the “housekeeping cells” in the CNS, which are the most abundant and play an important role in the protection of the CNS, and any dysfunction of astrocytes will seriously affect the survival of neuronal cells (Takuma et al., 2004). AQP4 is the most abundant aquaporin in the CNS, which is expressed in the perivascular areas of the BBB and the endfoot areas of astrocytes in the form of polarized distribution. It participates in the regulation of bidirectional water flow in the brain microenvironment and ensures the balance of water homeostasis in the brain (Ren et al., 2013). Under pathological conditions, the polar distribution of AQP4 is disrupted, resulting in a scattered distribution across the plasma membrane of astrocytes. This phenomenon, known as “depolarization,” exacerbates brain edema and triggers astrocyte activation, thereby mediating the progression of brain injury (Xin et al., 2023). In models of traumatic brain injury, targeting AQP4 has been shown to attenuate CNS edema for protection against brain injury (Xiong et al., 2021; Kitchen et al., 2020). However, there are no reports on the changes in AQP4 expression in RIBI models and whether it is involved in the pathologic development of RIBI.

In this study, we constructed a RIBI model by whole-brain radiation on SD rats, focusing on describing the effects of AQP4 on cerebral edema, BBB injury, inflammatory response and apoptosis, and attempting to reveal its potential mechanism of action. Our study demonstrated that in the rat RIBI model, the use of the specific inhibitor AER-271 during the acute phase inhibited the expression of AQP4, reduced the degree of cerebral edema, inhibited astrocyte activation, attenuated neuro-inflammation, maintained the integrity of the BBB, and attenuated apoptosis, leading to neuro-protective effects. AQP4 is expected to be a potential target for the treatment of RIBI and provides new ideas for our understanding of the pathogenesis of RIBI, while AER-271 is expected to be a drug candidate for the treatment of RIBI.

## 2 Materials and methods

### 2.1 Animals

Specific pathogen free (SPF) male SD rats (6 weeks old) with an average weight of 200 ± 18 g were used in the study. A total of 270 rats were obtained from the Laboratory Animal Center of Soochow University under license: SCXK (Jiangsu) 2022-0006. All animals were maintained in pathogen-free conditions (22°C ± 2°C, 40%–55% humidity, and a 12 h light-dark cycle) prior to the experiments. All experiments were performed in strict accordance with the UK Animals (Scientific Procedures) Act and associated guidelines and the 2013 AVMA Animal Euthanasia guidelines. All animal studies were reviewed and approved by the Ethics Committee of the Animal Center of Soochow University with ethics batch number (202401A1205).

### 2.2 Experimental grouping design

#### 2.2.1 Experimental grouping 1

**FIGURE 1:**
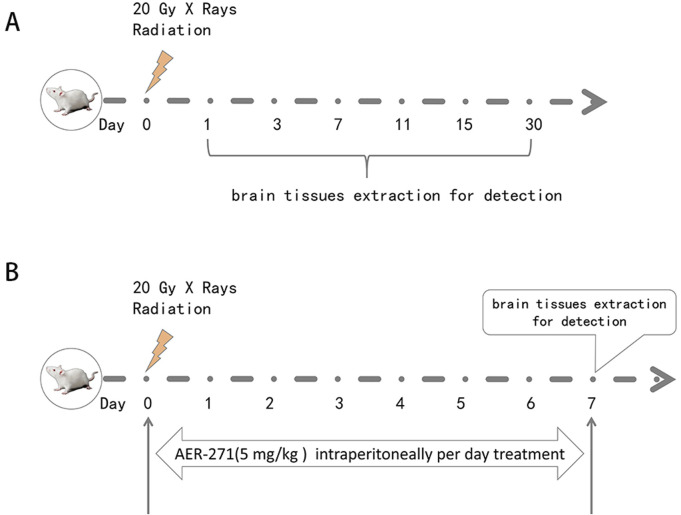
Schematic diagram of rat RIBI model construction, drug treatment and detection time. **(A)** Schematic diagram of rat RIBI model construction and detection time. **(B)** Flow chart of whole-brain radiation and drug therapy in rats.

110 SD rats were randomly and equally divided into Sham group (sham operation) and IR group (performed 20Gy X-rays whole brain radiation). Brain tissues were taken on the 1, 3, 7, 11, 15 and 30 d after radiation and examined, as shown in Figure 1A.

#### 2.2.2 Experimental grouping 2

160 SD rats were randomly and equally divided into Sham group (sham operation), AER-271 group (AER-271 treatment), IR group (20Gy X-rays radiation whole brain radiation) and IR+AER-271 group (20Gy X-rays whole brain radiation with simultaneous AER-271 treatment), the radiation and drug treatment process was shown in Figure 1B. Brain tissues were taken on day 7 for subsequent examined.

### 2.3 Radiation conditions

Animals were anesthetized with 1% pentobarbital (50 mg/kg) and then fixed in a prone position on a treatment bed before irradiation. The rest of the body was shielded with a lead block to expose only the brain. A medical linear accelerator (Siemens Mevatron MD2, Erlangen, Germany) was used to deliver 20Gy X-rays whole brain radiation at a dose rate of 2Gy/min with a source skin distance of 100 cm. The radiation dose of 20Gy was chosen based on previous studies that found that a single dose of 20Gy X-rays could successfully construct the RIBI model (Xu et al., 2021; Xiong et al., 2024). Radiation experiments were performed in the Department of Radiation Oncology, the First Affiliated Hospital of Soochow University.

### 2.4 Pharmacological interventions

AER-271 (HY-115460, MedChemExpress, New Jersey, United States) was dissolved in 20 mL using a diluent consisting of 10% dimethyl sulfoxide (DMSO) and 90% saline to obtain a stock solution with a concentration of 2.5 mg/mL. The stock solution was dispensed and stored at −80°C for subsequent use. Before each experiment, the reserve solution was diluted to a concentration of 0.25 mg/mL using saline (0.9% NaCl) to obtain a fresh working solution of AER-271. The therapeutic dose of AER-271 was 5 mg/kg, which, according to the study, was reported to be effective in decreasing the degree of cerebral edema after cardiac arrest in SD rats, resulting in a reduction of cerebral edema by 3 h 82.1% and 100% reduction in the degree of cerebral edema at 6 and 24 h (Wallisch et al., 2019). The final concentration of DMSO in the experimental mixture was 1.0%, which was determined to have no significant effect on the experimental results (Li and Zhao, 2007).

### 2.5 H&E staining

Brain tissues from rats were fixed in 4% paraformaldehyde for 24 h and then paraffin-embedded. Paraffin sections were made from 5 μm thick tissue. The sections were baked at 90°C for 10 mins, then dewaxed in xylene, then hydrated with gradient ethanol, then stained with hematoxylin (Beyotime, Jiangsu, China) for 10 min and eosin (Beyotime, Jiangsu, China) for 2 min, and then sealed with neutral gum. The sections were observed under a light microscope and photographed. The experiments were performed in three independent replicates and a total of 33 animals were used.

### 2.6 Brain water content

The water content of brain tissue was measured using the dry/wet weighing method to determine the extent of cerebral edema. After the animal were deeply anesthetized, the brain tissue vasculature is flushed with pre-cooled PBS to remove blood until a colorless fluid is obtained. The intact brain tissues were removed, filter paper was used to absorb the surface moisture and immediately weighed on an electronic analytical balance to obtain the wet weight (WW). The brain tissues were then dried in an oven (65°C) and weighed every 24 h until the difference between the two weights before and after was less than 1% to obtain the dry weight (DW). The brain water content was calculated as follows: (WW–DW)/WW × 100%. The experiments were performed in three independent replicates and a total of 33 animals were used.

### 2.7 EB assay

Rats were injected with 2% EB solution (Klamar-reagent, Shanghai, China) at a dose of 0.1 ml/100 g via the tail vein. After free movement for 1 h, the rats were anesthetized, and the precooled PBS was infused transcardially until the supernatant was drained. Brain tissues were taken, accurately weighed to obtain the wet weight (WW), cut into 1 mm^3^ size pieces and placed in EP tubes and soaked in 3 ml formamide reagent. The EP tubes were placed in a water bath at 37°C for 72 h, and centrifuged at 15,000 rpm for 15 min. The supernatant was taken and the absorbance in each EP tube was measured at 620 nm using an enzyme meter. EB content in brain tissue (µg/g) = brain tissue EB concentration (µg/ml) × 3 ml/WW (g). The experiments were performed in three independent replicates and a total of 33 animals were used.

### 2.8 Western blot

100 mg of brain tissue in the cerebral cortex was lysed with 1 ml of precooled RIPA protein lysate (Beyotime, Jiangsu, China) for 15 min on ice to extract protein, centrifuged at 12,000 rpm for 10 min at 4°C, and the supernatant was removed. Protein concentration was determined using a BCA kit (KeyGen BioTECH, Jiangsu, China). According to the molecular weight of the target protein, a suitable concentration of SDS-PAGE gel was selected for electrophoretic separation of the protein specimen, and the separated proteins were transferred to a PVDF membrane, and then the membrane was closed using 5% concentration of skimmed milk for 2 h at room temperature, the primary antibody was incubated at 4°C for 16 h, and washed three times with TBST, and the secondary antibody was incubated at room temperature for 2 h, and washed three times with TBST, and bands were visualized by using an ECL kit (Beyotime, Jiangsu, China) to display the bands, chemiluminescence gel imager (Carestream Health, Inc., Rochester, NY, United States) to observe the bands, and then use ImageJ software to analyze the gray value for quantitative analysis of protein expression. The primary antibodies used in this study were: Anti-AQP4 (#59678, 1:1,000, CST, Massachusetts, United States), Anti-GFAP (#3670, 1:1,000, CST, Massachusetts, United States), Anti-phosphorylated-STAT-3 (p-STAT3) (#9145, 1:1,000, CST, Massachusetts, United States), Anti-STAT3 (#9139, 1:1,000, CST, Massachusetts, United States), Anti-phosphorylated-janus kinase-2 (p-JAK2) (#3771, 1:1,000, CST, Massachusetts, United States), Anti-JAK2 (#3230, 1:1,000, CST, Massachusetts, United States), Anti-ZO-1 (A11417, 1:1,000, ABclonal, Wuhan, China), Anti-Occludin (#91131, 1:1,000, CST, Massachusetts, United States), Anti-Claudin-5 (ab172968, 1:1,000, Abcam, Cambridge, United Kingdom), Anti-phosphorylated-mTOR (p-mTOR) (#5536, 1:1,000, CST, Massachusetts, United States), Anti-mTOR (#2983, 1:1,000, CST, Massachusetts, United States), Anti-phosphorylated-PI3K (p-PI3K) (ab235266, 1:1,000, Abcam, Cambridge, United Kingdom), Anti-PI3K (ab302958, 1:1,000, Abcam, Cambridge, United Kingdom), Anti-phosphorylated-AKT (p-AKT) (#4060, 1:1,000, CST, Massachusetts, United States), Anti-AKT (#4691, 1:1,000, CST, Massachusetts, United States), Anti-ERK (#4695, 1:1,000, CST, Massachusetts, United States), Anti-Caspase3 (#9662,1:1,000, CST, Massachusetts, United States), Anti-Cleaved Caspase3 (#9664, 1:1,000, CST, Massachusetts, United States), Anti-GAPDH (#2118, 1:3,000, CST, Massachusetts, United States). The experiments were performed in three independent replicates and a total of 39 animals were used.

### 2.9 qRT-PCR

Total RNA from brain tissues in the cerebral cortex was extracted with Trizol reagent (Servicebio, Wuhan, China), and the ratio and concentration of A260/A280 were determined by NanoDrop spectrophotometer, and then the concentration was adjusted to 0.5–1.0 μg/μl with DEPC H_2_O. cDNA was synthesized using the TB Green Premix EX TaqTM II kit (Takara, Tokyo, Japan) to synthesize cDNA under the conditions of 37°C for 15 min, 85°C for 5 s, and 4°C for storage. Real-time PCR analysis was then performed. A 20 µl PCR reaction solution was prepared on ice according to the kit instructions and the PCR reaction was performed using an ABI 7500 Real-Time PCR System (Applied Biosystems, CA, United States). The program was 40 cycles of pre-denaturation at 95°C for 30 s, 95°C for 5 s and 60°C for 30 s. The PCR reaction was performed at the end of the cycle. A lysis curve was plotted at the end of the PCR cycle to verify the correct generation of PCR products. Gene expression was normalized to GAPDH and calculated using the C(t) method (see Table 1 for primer sequences, GAPDH was used as an internal reference). The experiments were performed in three independent replicates and a total of 30 animals were used.

**TABLE 1:** Primer pairs used for qRT-PCR.

Genes	Primer pairs	
AQP4	Forward (5′→3′)	GTC​CTC​ATC​TCC​CTC​TGC​TTT
	Reverse (5′→3′)	GAA​GAC​GGA​CTT​GGC​GAT​G
GFAP	Forward (5′→3′)	AAT​TGC​TGG​AGG​GCG​AAG​AA
	Reverse (5′→3′)	TTG​AGG​TGG​CCT​TCT​GAC​AC
IL-1β	Forward (5′→3′)	ATG​ATG​GCT​TAT​TAC​AGT​GGC​AA
	Reverse (5′→3′)	GTC​GGA​GAT​TCG​TAG​CTG​GA
IL-6	Forward (5′→3′)	GCC​CAC​CAG​GAA​CGA​AAG​TC
	Reverse (5′→3′)	TGG​CTG​GAA​GTC​TCT​TGC​G
IL-10	Forward (5′→3′)	AAT​TGA​ACC​ACC​CGG​CAT​CT
	Reverse (5′→3′)	TTT​CCA​AGG​AGT​TGC​TCC​CG
COX-2	Forward (5′→3′)	CGC​GCT​AGC​ATC​GAT​CAG​CTA​GC
	Reverse (5′→3′)	CGG​GCT​AGC​TAC​GAT​CGC​TAC​G
TNF-α	Forward (5′→3′)	GGA​GGG​AGA​ACA​GCA​ACT​CC
	Reverse (5′→3′)	GCC​AGT​GTA​TGA​GAG​GGA​CG
VEGFA	Forward (5′→3′)	CGG​GCC​TCT​GAA​ACC​ATG​AA
	Reverse (5′→3′)	GCT​TTC​TGC​TCC​CCT​TCT​GT
HIF-1α	Forward (5′→3′)	AGG​TTG​AGG​GAC​GGA​GAT​TT
	Reverse (5′→3′)	TGG​CTG​CAT​CTC​GAG​ACT​TT
ICAM-1	Forward (5′→3′)	TTT​TCA​GCT​CCC​ATC​CTG​ACC
	Reverse (5′→3′)	GGG​AAG​TAC​CCT​GTG​AGG​TG
GAPDH	Forward (5′→3′)	CCCTCTGGAAAGCTGTGG
	Reverse (5′→3′)	AGT​GGA​TGC​AGG​GAT​GAT​G

### 2.10 Elisa

The levels of interleukin-6 (IL-6) and tumor necrosis factor-a (TNF-α) in the cortical area of rat brain tissue were detected using Rat Elisa IL-6 Kit and Rat ELISA TNF-α Kit (COIBO BIO, Shanghai, China). A sample of 100 mg of cortical area of brain tissue was taken and then cut into 1 mm^3^ pieces of tissue, followed by the addition of pre-cooled 1× cell extraction buffer (COIBO BIO, Shanghai, China) (100 mg/1 ml), which was well homogenized using a tissue homogenizer, and then incubated on ice for 20 min, and the supernatant was taken after centrifugation at 15,000 rpm for 45 min. 50 μl of the supernatant of the sample to be detected was added to the detection wells, followed by the addition of 100 μl of horseradish peroxidase (HRP)-labeled detection antibody, and incubated for 60 min at 37°C in a temperature chamber. Next, the reaction solution was washed according to the kit instructions, and then the reaction solution was added and the final absorbance was measured at 450 nm using an enzyme meter to detect the cytokine content. The experiments were performed in three independent replicates and a total of 20 animals were used.

### 2.11 Immunofluorescence staining

Paraffin sections of 5 μm thick brain tissue were taken and baked at 65°C for 60 min, then the paraffin was removed with xylene and hydrated with gradient ethanol. Antigen repair was performed using sodium citrate antigen repair solution (Beyotime, Jiangsu, China) in a water bath at 100°C for 20 min, and the tissues were closed with immunostaining blocking solution (Beyotime, Jiangsu, China) at 37°C for 30 min, followed by overnight incubation at 4°C with the following primary antibodies. After washing with PBS, the tissues were incubated with Alexa-Fluor 488 and 555 donkey anti-rabbit/mouse secondary antibodies (Beyotime, Jiangsu, China) for 2 h at room temperature. Finally, the nuclei were stained and blocked with anti-fluorescence quenching sealer (containing DAPI) (Beyotime, Jiangsu, China). Images were taken by an Olympus confocal microscope (Olympus FV1200, Tokyo, Japan). The primary antibodies used in this study were: Anti-AQP4 (#59678, 1:200, CST, Massachusetts, United States), Anti-GFAP (#3670, 1:500, CST, Massachusetts, United States), Anti-ZO-1 (A11417, 1:100, ABclonal, Wuhan, China), Anti-Occludin (#91131, 1:500, CST, Massachusetts, United States), Anti-CD31 (28083-1-AP, 1:200, proteintech, Chicago, United States), Anti-Cleaved Caspase3 (#9664, 1:200, CST, Massachusetts, United States). The experiments were performed in three independent replicates and a total of 82 animals were used.

### 2.12 CCK8

The toxic effects of different concentrations of AER-271 on rat astrocytes were detected by CCK-8 assay. Astrocytes in logarithmic growth phase were taken and inoculated into 96-well plates at 1*10^5^/100 μl per well, and the AER-271 working solution was diluted with serum-free medium so that the final concentrations were (2, 5, 10, 20, 40 μM), and then cultured in a 37°C cell After 24 h of incubation in 37°C, 10 μl of CCK-8 solution (Beyotime, Shanghai, China) was added to each well and incubated at 37°C for 2 h. The absorbance was measured at 450 nm using an enzyme marker to calculate the cell proliferation viability.

### 2.13 Flow cytometry

Rat astrocytes were cultured in DMEM medium (Gibco, Carlsbad, CA, United States) supplemented with 10% fetal bovine serum (Gibco, Carlsbad, CA, United States), 1% sodium penicillin and 100 mg/mL streptomycin (Beyotime, Shanghai, China) in a cell culture incubator at 37°C containing 5% CO_2_. Cells in logarithmic growth phase were taken and divided into Sham group (no treatment), AER-271 group (addition of AER-271 working solution at a final concentration of 5 μM), IR group (20Gy X-ray single radiation), and IR+AER-271 group (addition of AER-271 working solution at a final concentration of 5 μM, and also 20Gy X-ray single radiation). Radiation was performed using a biological X-ray irradiator (Rad source RS-2000 Pro, United States) with an energy of 160 kv, a dose rate of 1.214 Gy/min, a field size of 20–30 cm, and a focus-surface distance of 20 cm, and the cellular radiation was carried out at the Radiation Center of Soochow University.

The cells were treated according to the experimental conditions for 24 h and then flow cytometry was performed. First, the cell culture solution was aspirated into a clean centrifuge tube, the cells were washed once with PBS, appropriate amount of trypsin cell digest (without EDTA) was added to digest the cells, the aspirated culture solution was added, and the cells were collected by centrifugation at 1,000 rpm for 5 min, the supernatant was discarded, and the cells were collected, resuspended in PBS and counted. Take 5–100,000 resuspended cells, centrifuge at 1,000 rpm for 5 min, discard the supernatant, and add 195 μl Annexin V-FITC conjugate to gently resuspend the cells. Add 5 μl Annexin V-FITC (Beyotime, Shanghai, China) and mix gently. Add 10 μl of propidium iodide (PI) (Beyotime, Shanghai, China) staining solution and mix gently. Incubate for 10–20 min at room temperature away from light, followed by placing in an ice bath, and then assayed using a flow cytometer (BD FACSVerse, United States).

### 2.14 Statistical analysis

Data were statistically analyzed using SPSS 23.0 statistical software. Graphs were plotted using GraphPad Prism 9.5. Data for continuous variables were expressed as mean ± standard deviation (x ± s). The t-test was used to estimate the differences between the two groups, and ANOVA and Tukey’s multiple comparison test were used to assess the differences between the four groups. P < 0.05 or 0.01 or 0.001 were considered statistically significant.

## 3 Results

### 3.1 The trends of AQP4 changes in the rat RIBI model and between AQP4 and cerebral edema

**FIGURE 2:**
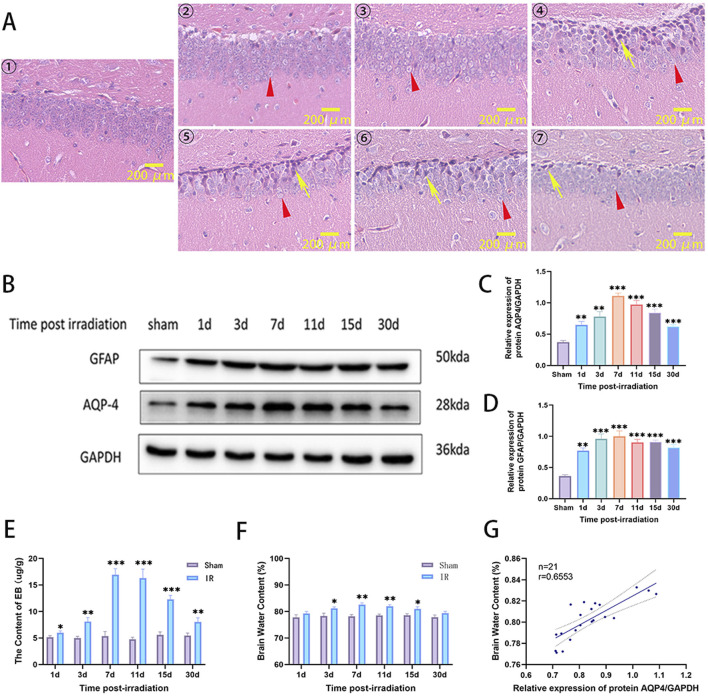
The changes of neuronal cell pathological damage, AQP4 and GFAP expression, brain tissue water content, and BBB permeability in rat RIBI model. **(A)** Representative images of H&E staining in the hippocampus of the rat RIBI model at different observation time points (①Sham group, ②-⑦ were 1, 3, 7, 11, 15, and 30 d post-irradiation. Triangles (red) show edematous cells, and arrows (yellow) show cells with deeply stained nuclei and nuclear consolidation. Scale bar: 200 μm). **(B)** The trends of protein expression of AQP4 and GFAP in rat RIBI model detected by Western blot. **(C)** In the RIBI rat model, AQP4 protein expression gradually increased from the first day after radiation, reached the peak on the seventh day, and then slowly decreased, and was still higher than that of the Sham group on the 30th day (n = 3). **(D)** In the RIBI rat model, the expression of GFAP protein increased significantly from the first day after radiation, reached the peak at the seventh day, then decreased slowly, and was still significantly higher than that of the Sham group at the 30th day (n = 3). **(E)** Changes of brain water content in RIBI rats (n = 3). **(F)** Changes of Evans blue permeability in the brain of rats with RIBI (n = 3). **(G)** Correlation analysis between AQP4 protein expression level and brain water content in rat RIBI model. There was a positive correlation between brain water content and AQP4 protein expression. (Y = 0.1370*X + 0.6877, r = 0.6553, n = 21). *P < 0.05, **P < 0.01, ***P < 0.001.

A RIBI model was constructed by whole-brain radiation of 20Gy X-rays to SD rats. The pathological changes of hippocampal neurons were detected by H&E staining at 1, 3, 7, 11, 15, and 30 d after radiation. The results showed that the edema of neuronal cells could be observed on the first day after radiation, and then the number of edematous cells gradually increased and reached the maximum on the seventh day, and then began to fade away slowly and basically disappeared on the 30th day. A large number of neurons with deep nuclear staining could be observed at 7 days after radiation, and then gradually decreased, and only a small number of neurons with deep nuclear staining could be observed at 30 days after radiation (Figure 2A). Western blot was used to detect the changes in the expression of GFAP and AQP4 in the brain tissue of rats. The results showed that the protein expression of GFAP and AQP4 increased on the first day after radiation, reached the highest level on the seventh day, then slowly decreased, and was still higher than that of the Sham group on the 30th day (Figures 2B–D). The integrity of BBB was evaluated by measuring the content of EB in brain tissue. The results showed that the content of EB in brain tissue increased significantly from the first day after radiation, reached the highest level on the seventh day, and then slowly decreased, and was still higher than that of the Sham group on the 30th day (Figure 2E). The effect of radiation on cerebral edema was assessed by measuring the water content of brain tissue, and the results showed that an increase in cerebral edema could be observed on day 1 after radiation, with a statistically significant difference on day 3, peaking on day 7, after which the cerebral edema began to decrease to a level similar to that of the Sham group by day 30 (Figure 2F). The correlation between AQP4 expression and cerebral edema was assessed by correlation analysis using SPSS software, which showed that the expression level of AQP-4 was positively correlated with the severity of cerebral edema (Figure 2G). These results suggest that in the model of RIBI, radiation promotes the expression of AQP4 and the expression level is higher in the acute phase (within 2 weeks after radiation), and the expression of AQP4 correlates with cerebral edema.

### 3.2 Radiation promoted AQP4 expression in rat brain tissue while disrupting the polar distribution state

**FIGURE 3:**
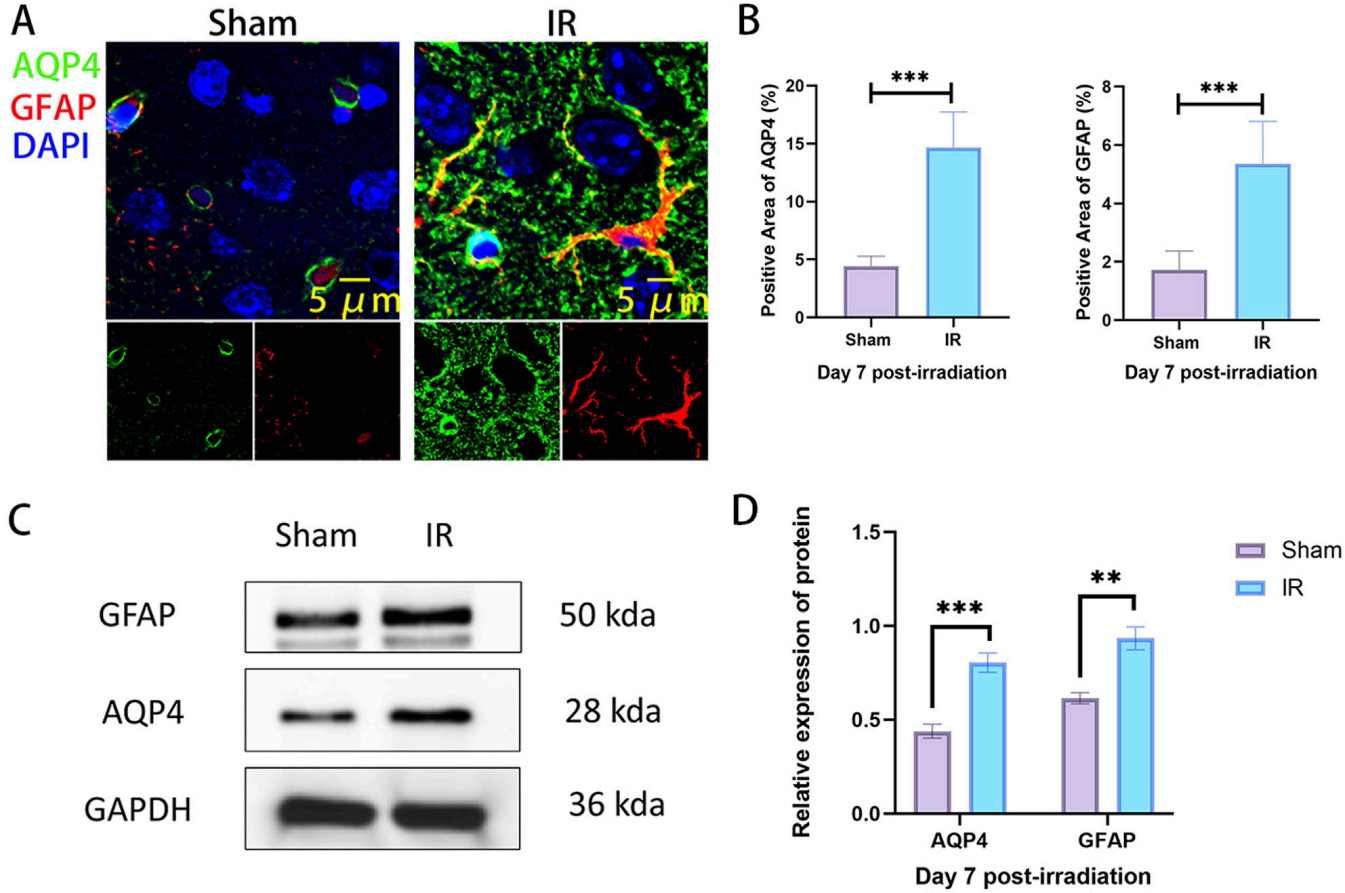
The expression of AQP4 is increased and the polarity distribution is changed in rat RIBI model. **(A)** Representative images of immunofluorescence staining for co-localized expression of AQP4 (green) and GFAP (red) in the rat RIBI model (Scale bar: 5 μm). **(B)** The expression areas of AQP4 and GFAP were significantly increased in the rat RIBI model (n = 5). **(C)** Western blot detection of protein expression of GFAP and AQP4 in rat RIBI model. **(D)** In the RIBI model, GFAP and AQP4 protein expression was significantly increased (n = 3). **P < 0.01, ***P < 0.001.

The previous study found that the most severe injury time point was at day 7 after radiation in the rat RIBI model. Subsequently, we examined the co-localized expression of AQP4 and GFAP in the brain tissue of Sham and IR groups on day 7 by immunofluorescence co-localization staining. The results showed that the area of GFAP expression was significantly increased in the IR group compared to the Sham group, suggesting that radiation caused the activation of astrocytes. In the Sham group, AQP4 was mainly distributed in the end-foot region of astrocytes as an aggregate, while in the IR group, AQP4 was decreased in the acropodia region of astrocytes as an aggregate and increased mainly in the plasma membrane region of astrocytes as a punctate distribution. At the same time, the co-localized expression of AQP4 and GFAP was also significantly increased (Figures 3A, B). This suggests that radiation promotes the expression of AQP4 in brain tissue and leads to “depolarization” of the distribution of AQP4. Western blot results also confirm that radiation promotes the increased expression of AQP4 and GFAP in rat brain tissue (Figures 3C, D).

### 3.3 Treatment with AER-271 attenuated cerebral edema, astrocyte activation and neuronal cell damage in the hippocampus in rats with RIBI

**FIGURE 4:**
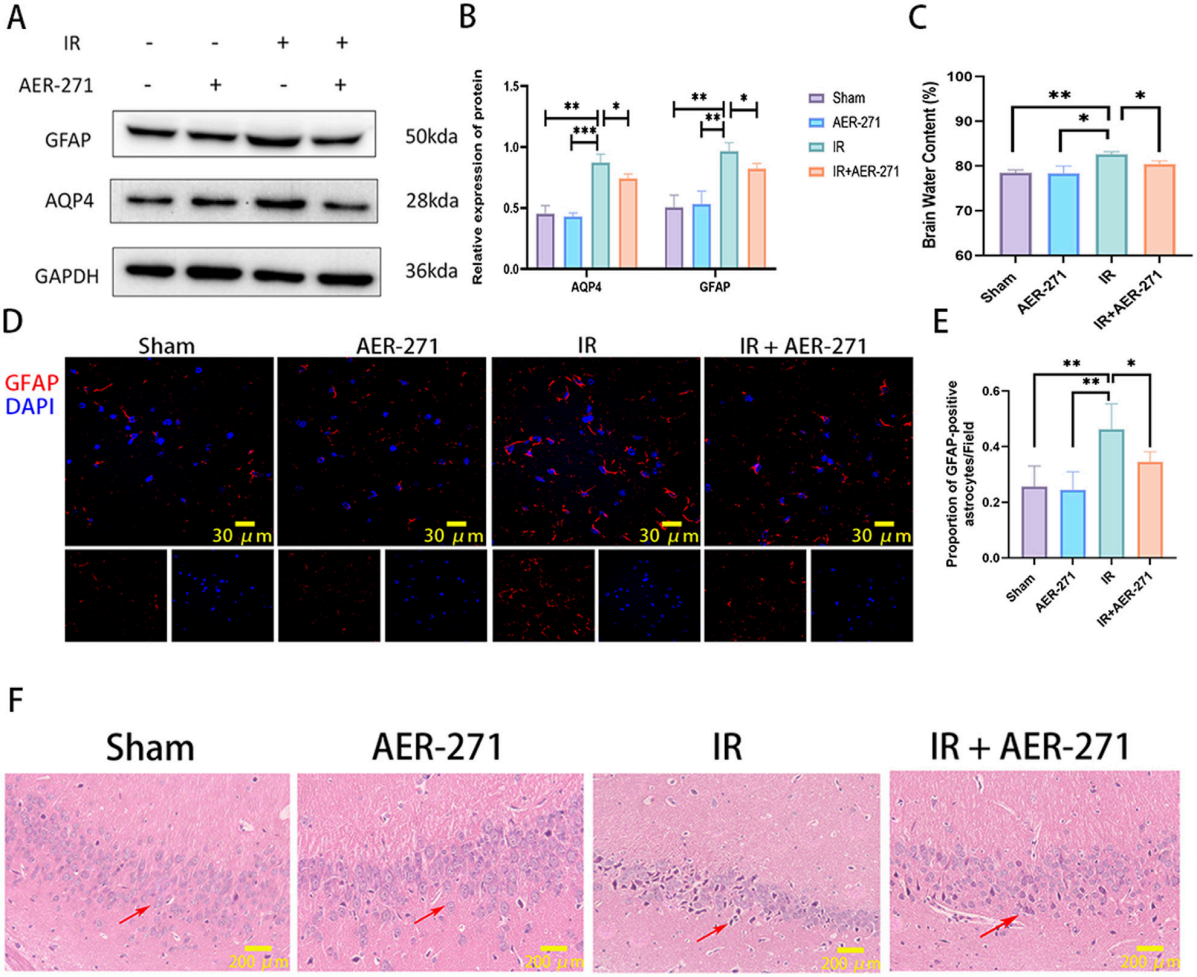
Treatment with AER-271 attenuated cerebral edema, astrocyte activation, and neuronal cell damage in the hippocampus. **(A)** Western blot detection of AQP4 and GFAP protein expression in brain tissues of rats from four treatment groups. **(B)** Radiation promoted AQP4 and GFAP protein expression in rat brain tissue, and treatment with AER-271 inhibited this promotion (n = 3). **(C)** Radiation promoted elevated brain tissue water content in rats and treatment with AER-271 attenuated radiation-induced cerebral edema (n = 3). **(D)** Representative images of GFAP (red) expression in cortical areas of rat brain tissue from four treatment groups detected by immunofluorescence staining (Scale bar: 30 μm). **(E)** Radiation resulted in a significant increase in the number of activated astrocytes in the cortical areas of rat brain tissue, and treatment with AER-271 attenuated radiation-induced astrocyte activation (n = 5). **(F)** H&E staining was used to detect neuronal cell damage in the hippocampal region of rat brain tissue in the four treatment groups. Radiation induced a pathologic damage in the hippocampal region of neuronal cells with deeply stained nuclei, and treatment with AER-271 attenuated the radiation-induced neuronal cell damage. The arrows show the diseased neuronal cells (Scale bar: 200 μm). *P < 0.05, **P < 0.01, ***P < 0.001.

To explore the protective effect of AER-271 on RIBI, rats were randomly divided into four treatment groups as described previously and examined on day 7 after radiation. First, we detected AQP4 and GFAP protein expression in the four treatment groups by Western blot, and the results showed that AER-271 treatment could inhibit the radiation-induced expression of AQP4 and GFAP *in vivo* (Figures 4A, B). AQP4 is a key protein regulating water balance in the brain (Szczygielski et al., 2021). We next examined whether treatment with AER-271 could reduce radiation-induced brain edema. As shown in Figure 4C, brain water content in the IR group was significantly higher than that in the Sham group and AER-271 group. However, the water content of the brain tissue in the IR+AER-271 group was significantly lower than that in the IR group, suggesting that AER-271 could attenuate radiation-induced brain edema by inhibiting AQP4 expression. The activation of astrocytes in the cortex is one of the important mechanisms mediating the inflammatory response in RIBI (Piskunov et al., 2015). We examined the effect of AER-271 treatment on astrocyte activation by immunofluorescence staining. The results showed that the number of GFAP-positive astrocytes in the cortex of rat brain tissue was significantly increased in the RIBI model. However, treatment with AER-271 reduced the number of radiation-induced activated astrocytes (Figures 4D, E). The damage of hippocampal neurons in the acute phase of RIBI is the main factor leading to late cognitive dysfunction (Rübe et al., 2023). We further examined the effect of AER-271 treatment on pathological damage of hippocampal neurons by H&E staining. As shown in Figure 4F, hippocampal neurons in the IR group showed a large number of nuclear dark staining changes. However, the number of neuronal cells with the above pathological damage was significantly reduced in the IR+AER-271 group, indicating that the treatment of AER-271 has a radioprotective effect on neuronal cells. Together, these results show that treatment with AER-271 exerts a neuro-protective effect on RIBI by inhibiting AQP4 expression.

### 3.4 Treatment with AER-271 attenuated the inflammatory response in RIBI

**FIGURE 5:**
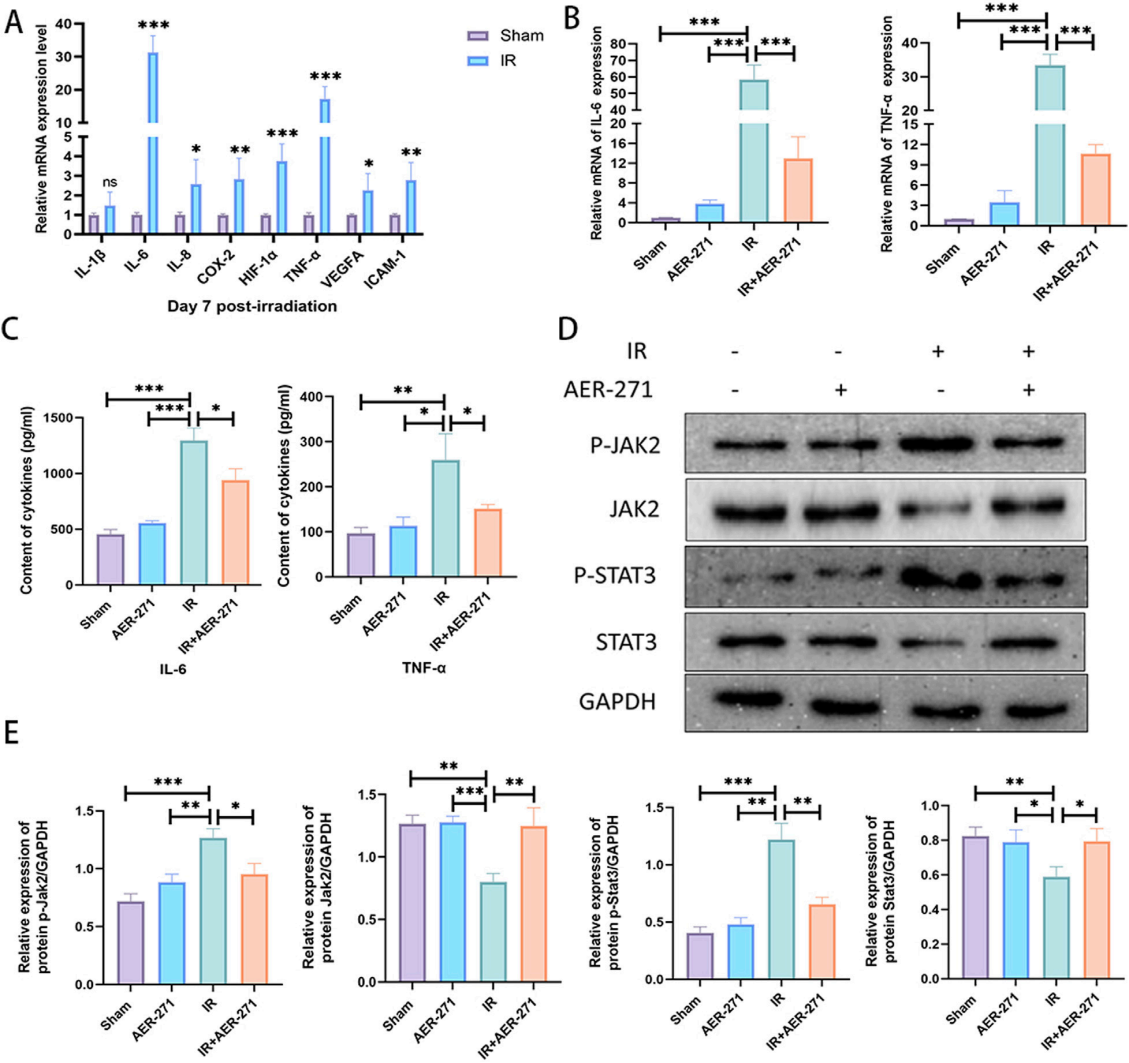
Treatment with AER-271 attenuated the inflammatory response in rat RIBI. **(A)** Radiation promoted the expression of inflammatory factors (IL-6, IL-8, COX-2, HIF-1α, TNF-α, VEGFA, ICAM-1) in rat brain tissue (n = 5). **(B)** Treatment with AER-271 attenuated radiation-induced IL-6 and TNF-α expression (n = 5). **(C)** Treatment with AER-271 attenuated radiation-induced IL-6 and TNF-α secretion (n = 5). **(D)** Western blot to detect the expression of proteins related to Jak2/Stat3 signaling pathway. **(E)** Radiation promoted the phosphorylated expression of JAK2 and STAT3, and treatment with AER-271 inhibited this promotion (n = 3). *P < 0.05, **P < 0.01, ***P < 0.001.

To investigate the effect of AER-271 treatment in inflammatory injury in RIBI. First, we examined the expression of some common inflammatory factors such as: interleukin-1β (IL-1β), IL-6, IL-8, cyclooxygenase-2 (COX-2), hypoxia-inducible factor-1α (HIF-1α), TNF-α, vascular endothelial growth factor-A (VEGFA), intercellular adhesion molecule-1 (ICAM-1), which have been reported to be involved in the development of RIBI and would mediate subsequent delayed injury, in a RIBI model by qRT-PCR (Yang et al., 2017; Wang et al., 2021). As shown in Figure 5A, the expression of IL-6, IL-8, COX-2, HIF-1α, TNF-α, VEGFA, and ICAM-1 were significantly increased in the rat RIBI model, among which the expressions of IL-6 and TNF-α were increased most significantly. Next, we examined the effect of AER-271 treatment on the expression and secretion of IL-6 and TNF-α, and the results showed that radiation significantly induced the expression and secretion of IL-6 and TNF-α in rat brain tissue, while AER-271 treatment inhibited this promoting effect (Figures 5B, C). To explore the mechanism of AER-271 treatment in RIBI inflammatory response, we examined the expression levels of proteins related to JAK2/STAT3 signaling pathway, which is downstream of IL-6, by Western blot (Neurath and Finotto, 2011). The results showed that the expression levels of JAK2 and STAT3 were decreased, while the expression levels of p-JAK2 and p-STAT3 were significantly increased in IR group compared with Sham group and AER-271 group. The expression levels of JAK2 and STAT3 were restored in IR+AER-271 group compared with IR group. The expression levels of p-JAK2 and p-STAT3 were reduced, suggesting that radiation promoted the phosphorylation of the JAK2/STAT3 signaling pathway and that treatment with AER-271 inhibited this promotion (Figures 5D, E). These results suggested that treatment with AER-271 attenuated the expression and release of inflammatory cytokines in the RIBI model by inhibiting AQP4 expression, and alleviated the inflammatory response by inhibiting the phosphorylation expression of JAK2/STAT3 pathway.

### 3.5 Treatment with AER-271 maintained the integrity of the blood-brain barrier in RIBI

**FIGURE 6:**
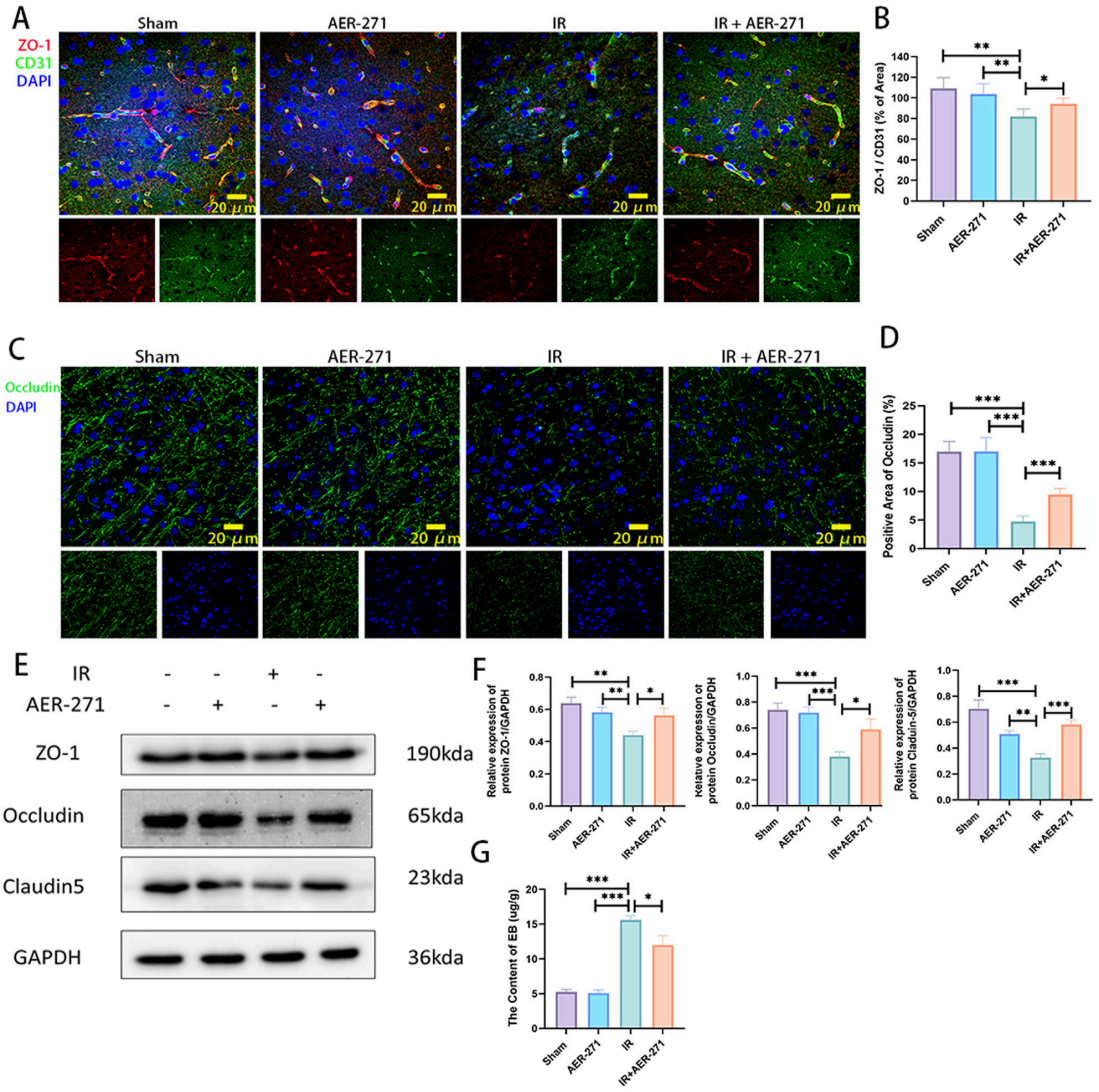
Treatment with AER-271 maintained the integrity of the BBB in rat RIBI. **(A)** Representative images of immunofluorescence co-localization staining for detection of tight junction protein ZO-1 (red) and vascular endothelial cell marker protein CD31 (green) (Scale bar: 20 μm). **(B)** Radiation inhibited the co-expression area of ZO-1 and CD31, and treatment with AER-271 restored the co-expression area of ZO-1 and CD31 (n = 5). **(C)** Representative images of the areas of membrane closure protein Occludin (green) expression detected by immunofluorescence staining. (Scale bar: 20 μm). **(D)** Radiation inhibited the expression region of the membrane closure protein Occludin, which was restored by treatment with AER-271 (n = 5). **(E)** Western blot detection of ZO-1, Occludin, and Claudin-5 expression in the brain tissues of rats in four treatment groups. **(F)** Radiation inhibited the expression of ZO-1, Occludin, and Claudin-5, while treatment with AER-271 restored their expression (n = 3). **(G)** The permeability of the BBB was evaluated by detecting the content of EB in brain tissue of rats in the four treatment groups. Radiation increased the permeability of the BBB, and AER-271 treatment decreased the permeability of the BBB (n = 3). *P < 0.05, **P < 0.01, ***P < 0.001.

We further investigated the effect of treatment with AER-271 on the integrity of the blood-brain barrier in RIBI. The blood-brain barrier is a dense barrier structure composed of continuous vascular endothelial cells and tight intercellular junctions, intact basement membranes, and endfeet of astrocytes (Abbott et al., 2010). Density of vascular endothelial cells, expression of tight junction proteins and occludin proteins are critical for maintaining the integrity of the BBB (Nordal and Wong, 2005; Zhang et al., 2024). Firstly, co-expression of tight junction protein ZO-1 and vascular endothelial cell marker CD31 was examined by immunofluorescence co-expression staining. As shown in Figures 6A, B, radiation inhibited the co-expression of ZO-1 and CD31 compared with the Sham and AER-271 groups, and this inhibitory effect leads to disruption of tight junctions between vascular endothelial cells, increased cellular gaps, and increased permeability. However, the co-expression of ZO-1 and CD31 was partially restored in the IR+AER-271 group, suggesting that the tightness of the blood-brain barrier could be restored by treatment with AER-271. We further detected the expression of Occludin in the brain tissue of the four treatment groups by immunofluorescence staining. In the Sham group and AER-271 group, Occludin was widely expressed around the cells and showed filamentous continuity, while in the IR group, the expression range of Occludin was significantly detected. In addition, the continuity of Occludin was destroyed, while in IR+AER-271 group, the expression area and continuity of occludin were observed to be restored to a certain extent (Figures 6C, D). We further examined the protein expression levels of ZO-1, Occludin and Claudin-5 in the brain tissue of the four treatment groups by Western blot, and the results also showed that radiation inhibited the expression of ZO-1, Occludin and Claudin-5. However, treatment with AER-271 attenuated the inhibitory effect of radiation (Figures 6E, F). Finally, we evaluated the permeability of the BBB by measuring the content of EB in brain tissue. As shown in Figure 6G, radiation caused a significant increase in the content of EB in brain tissue, while treatment with AER-271 reduced this promoting effect. The results of the above experiments suggest that treatment with AER-271 can maintain the integrity of the BBB in RIBI.

### 3.6 Treatment with AER-271 reduced apoptosis in RIBI

**FIGURE 7:**
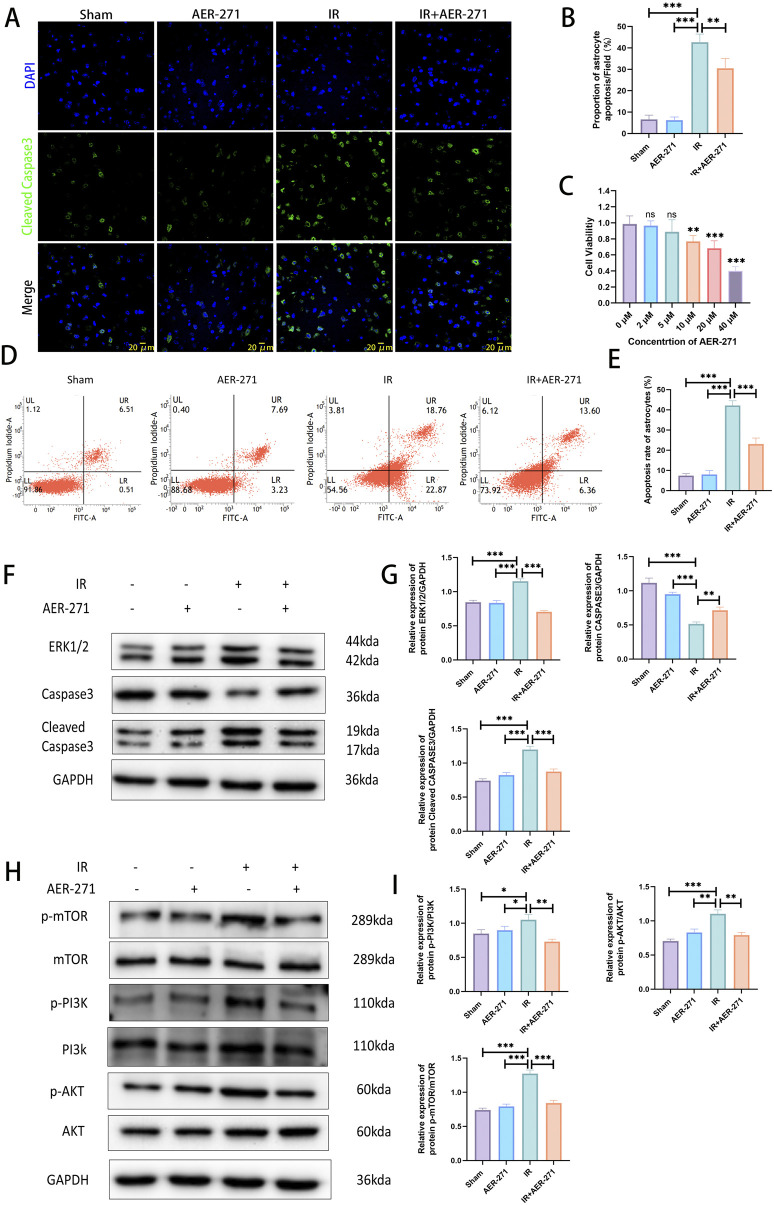
Treatment with AER-271 reduces apoptosis in rat RIBI. **(A)** Representative images of immunofluorescence staining to detect Cleaved Caspase3 expression in the brain tissues of rats from four treatment groups (Scale bar: 20 μm). **(B)** Radiation increased the number of Cleaved Caspase3-positive cells and promoted apoptosis, and treatment with AER-271 reduced the number of radiation-induced apoptotic cells (n = 5). **(C)** Cell viability assay of different concentrations of AER-271 treatment (2, 5, 10, 20, 40 μM) for 24 h to test cytotoxicity of AER-271 on astrocytes (n = 5). **(D)** Flow cytometry detection of astrocyte apoptosis in four treatment groups. **(E)** *In vitro*, radiation significantly increased astrocyte cell apoptosis, whereas treatment with AER-271 reduced radiation-induced astrocyte apoptosis (n = 5). **(F)** Western blot Detection of Apoptosis-Related Protein Expression in Brain Tissues of Rats in Four Treatment Groups. **(G)** Radiation promoted ERK expression in rat brain tissue and facilitated the conversion of Caspase3 to Cleaved Casapase3, which was inhibited by treatment with AER-271 (n = 3). **(H)** Western blot detection of phosphorylated expression of PI3K/AKT/mTOR signaling pathway in brain tissues of tats in four treatment groups. **(I)** Radiation promoted the phosphorylated expression of the PI3K/AKT/mTOR signaling pathway in rat brain tissue, while AER-271 treatment inhibited this promotion (n = 3). *P < 0.05, **P < 0.01, ***P < 0.001.

To investigate the effect of AER-271 treatment on RIBI cell apoptosis and its mechanism. We first detected the number of apoptotic cells in rat brain tissues of the four treatment groups by immunofluorescence staining, and the results showed that the proportion of Cleaved Caspase3-positive cells in the IR group was significantly higher than that in the Sham group and the AER-271 group, which indicated that radiation led to an increase in the number of apoptotic cells in the brain tissues of the rats, while the treatment of AER-271 reduced the radiation-induced apoptosis (Figures 7A, B). Astrocytes are the most abundant cells in rat brain tissue, and we next verified the radio-protective effects of AER-271 in in vitro experiments. First, we screened a 5 μM concentration of AER-271 for subsequent experiments by CCK8 assay (Figure 7C), and then detected the apoptosis rate of astrocytes in the four treatment groups by flow cytometry, which showed that the apoptosis rate of astrocytes in the IR group was much higher than that in the Sham group and the AER-271 group, which indicated that *in vitro*, radiation also significantly induced astrocyte apoptosis, while the apoptosis rate of astrocytes in the IR+AER-271 group was significantly lower than that in the IR group, suggesting that treatment with AER-271 could inhibit the induction of astrocyte apoptosis by radiation (Figures 7D, E). Next, we detected the expression of apoptosis-related marker proteins ERK, Caspase3 and Cleaved Caspase3 by Western blot. The results showed that radiation promoted the expression of ERK and the conversion of Caspase3 to Cleaved Caspase3 in the IR group compared with the Sham and AER-271 group, suggesting that radiation promoted apoptosis within rat brain tissue, which was reversed in the IR+AER-271 group (Figures 7F, G), suggesting that AER -271 treatment could attenuate radiation-induced apoptosis. To clarify the mechanism of AER-271 treatment in regulating apoptosis, the expression of PI3K/AKT/mTOR, the classical signaling pathway of apoptosis regulation, was detected by Western blot. The results showed that the phosphorylation levels of PI3K, AKT and mTOR in the IR group were significantly higher than those in the Sham group and AER-271 group, indicating that radiation activated the PI3K/AKT/mTOR signaling pathway. However, treatment with AER-271 reduced the phosphorylation of PI3K, AKT and mTOR, and inhibited the activation of PI3K/AKT/mTOR pathway (Figures 7H, I). The above results suggest that treatment with AER-271 can attenuate apoptosis in RIBI, and this may be achieved by regulating the PI3K/AKT/mTOR signaling pathway.

## 4 Discussion

The pathogenesis of RIBI is not yet fully understood, and the known view is that it is the result of a combination of radiation-induced DNA double-strand breaks, inflammatory response, oxidative stress, cellular structure disruption, blood-brain barrier disruption, and apoptosis (Gan et al., 2023). There are limited therapeutic options for RIBI. Steroids provide rapid symptomatic relief of tumor-related edema and radionecrotic edema, but side effects are also well defined. Other alternative drugs such as bevacizumab, mannitol, adrenocorticotropin, and boswellic acids have been used, but various barriers to further implementation remain, and these therapies are mainly symptomatic, with general overall effectiveness and side effects, and should not be applied long-term (Zhuang et al., 2019; Voss et al., 2021; Goldman et al., 2022). Therefore, the search for new therapeutic targets is important for understanding the pathogenesis of RIBI and finding potential therapeutic agents.

AQP4 is the most abundant aquaporin in the CNS, and studies have found that inhibition of AQP4 expression can reduce the degree of cerebral edema, inflammatory response and apoptosis in traumatic brain injury (Xing et al., 2023; Lu Q. et al., 2022). In AQP4-deficient mice, hypoglycemia-induced secretion of pro-inflammatory factors, the degree of inflammatory response and the degree of BBB destruction were significantly lower than those in normal mice (Zhao et al., 2018). In the present study, we found that in the rat RIBI model, radiation promoted the expression of AQP4 (Figures 2B, C), and the expression level of AQP4 was linearly correlated with the severity of cerebral edema (Figure 2G), and radiation also induced a “depolarization” of AQP4 distribution (Figure 3A). This change results in the inability of cerebral interstitial fluid to return to the veins through the lymphoid system, promoting cerebral edema (Liu et al., 2021; Sun et al., 2022; Zhu et al., 2022). This finding suggests that AQP4 is involved in the pathogenesis of RIBI and is expected to be a potential target for the treatment of RIBI.

We chose to use AER-271 to inhibit the expression of AQP4 to assess the protective effect and mechanism of action of AER-271 in RIBI. Cerebral edema can be divided into cytotoxic edema and vasogenic edema, both of which are regulated by AQP4 (Szlufik et al., 2024). Previous studies have found that radiation can cause cerebral vasculopathy (Kralik et al., 2017), loss of vascular endothelial cells (Mao et al., 2010), and mediate the development of vasogenic edema. In this study, we found that radiation disrupts the polarized state of AQP4 polymer distribution and promotes its expression in the plasma membrane region of astrocytes in a dotted-distribution pattern (Figure 3A), and this increase in abundance at the cell surface promotes the entry of water molecules into astrocytes, leading to cellular edema (Kitchen et al., 2020). And the degree of cerebral edema in RIBI could be reduced by treatment with AER-271 (Figure 4C), which further confirms that AQP4 plays an important role in the cerebral edema link in RIBI.

In addition, we found that treatment with AER-271 effectively suppressed astrocyte activation in RIBI (Figures 4D, E). Astrocyte activation has been reported to secrete large amounts of pro-inflammatory factors and many other cytokines that mediate inflammatory and remodeling processes (Glass et al., 2010). The accumulation of these inflammatory factors changes the brain microenvironment into a pro-inflammatory environment, leading to the occurrence of subsequent injury events. In this study, we found that the expression of multiple inflammatory factors was significantly elevated in the RIBI model (Figure 5A), and we focused on IL-6 and TNF-α. Treatment with AER-271 inhibited the expression and secretion of IL-6 and TNF-α in the cortical area of rats in the RIBI model (Figures 5B, C). Studies have shown that TNF-α can be first expressed in astrocytes after radiation and then released in large quantities, acting directly or indirectly on neuronal cells and microglia, activating the nuclear transcription factor-κB (NF-κB) signaling pathway, and inducing the expression of inflammatory factors, such as IL-6, IL-8, and IL-10, ICAM-1, and others (Wang et al., 2021; Chen et al., 2019; Zhang et al., 2021). These inflammatory factors can cause leukocyte adhesion, cell swelling, capillary dilation, reactive astrocyte proliferation and blood-brain barrier disruption (Zhang et al., 2021; Lu H. et al., 2022). A maintained state of inflammatory factors can lead to sustained injury in the CNS, and the mechanism of action may be closely related to the activation of the JAK2/STAT3 signaling pathway (Wu et al., 2020; Zhou et al., 2020). JAK2/STAT3 signaling pathway is an important component of multiple cytokine receptor systems, such as IL-6, which is involved in the regulation of cell growth, proliferation, differentiation and pathogen resistance. Within the CNS, the JAK2/STAT3 signaling pathway can be activated in response to stimulus conditions, such as ischemia, trauma, and radiation, and is involved in the regulation of apoptosis, inflammatory responses, vascular remodeling, oxidative stress, and cellular autophagy (Li H. et al., 2003; Zhu et al., 2021; Zhong et al., 2022). In the present study, radiation activated the JAK2/STAT3 signaling pathway by promoting the expression and release of inflammatory factors in the CNS and promoted the level of JAK2 and STAT3 phosphorylation expression, whereas treatment with AER-271 inhibited the release of inflammatory factors by suppressing the expression of AQP4 and suppressed the expression of JAK2 and STAT3 phosphorylation (Figures 5D, E), exerted a cerebroprotective effect.

The BBB is formed by vascular endothelial cells, astrocytes, and pericytes through tight junction proteins to form a dense barrier, the integrity of which is critical for maintaining homeostasis in the brain (Abbott et al., 2010). In the present study, we found that radiation inhibited the co-expression of the tight junction protein ZO-1 and the vascular endothelial cell marker protein CD31 (Figures 6A, B), as well as inhibited the expression region of the membrane closure protein Occludin (Figures 6C, D), and the expression of the tight junction protein Claudin-5 was significantly reduced (Figures 6E, F), while treatment with AER-271 attenuated the inhibitory effect of radiation on tight junction proteins and decreased the permeability of the blood-brain barrier (Figure 6G). Inhibition of AQP4 expression has also been found to attenuate blood-brain barrier disruption in other brain injury models (Lu Q. et al., 2022; Liu et al., 2020; Wang et al., 2024), which is consistent with our findings. It has been reported that in the CNS of rodents, apoptosis has been reported to occur as early as 3–4 h after radiation, and affected cells include astrocytes, oligodendrocytes, subventricular cells, neuronal cells, vascular endothelial cells, and neural precursor cells in the hippocampal region (Li et al., 1996; Shinohara et al., 1997; Li Y. Q. et al., 2003). The subsequent apoptosis is associated with neuroinflammation and the perturbation of some signal transduction. Our study found that AER-271 treatment attenuated apoptosis in the RIBI model (Figures 7A–D). AQP4 has been found to be a mediator in the pathogenesis of brain-related diseases and has a dual role in apoptosis signaling regulation by modulating processes related to brain edema (Dadgostar et al., 2021; Peng et al., 2023). In traumatic brain injury, inhibition of AQP4 expression reduces apoptosis (Goldman et al., 2022), and in neuroinflammation, AQP4 overexpression exacerbates astrocyte edema and apoptosis (Lu H. et al., 2022), which is consistent with our findings. The PI3K/AKT/mTOR pathway plays a crucial role in many physiological processes in organisms, and several studies have reported that the PI3K/AKT/mTOR pathway is involved in the regulation of apoptosis in the CNS (Guan et al., 2023; Li et al., 2023; Gao et al., 2023). In this study, radiation promoted the phosphorylated expression of the PI3K/AKT/mTOR signaling pathway, whereas treatment with AER-271 inhibited the phosphorylated activation of the PI3K/AKT/mTOR signaling pathway (Figures 7E, F), suggesting to us that in the pathogenesis of RIBI, AQP4 may be the PI3K/AKT/mTOR signaling pathway’s upstream target.

## 5 Conclusion

In summary, the present study highlights the role of AQP4 in the acute phase of RIBI, which participates in the regulation of subsequent events such as inflammatory response, BBB damage, and apoptosis by mediating the onset of brain edema, which provides a new perspective for our understanding of the pathogenesis of RIBI. And by attenuating cerebral edema, neuronal cell injury, inflammatory response, blood-brain barrier disruption and apoptosis in RIBI through the treatment of AER-271, AER-271 is expected to be a candidate drug for the treatment of RIBI. Of course, there are some limitations in this study. Such as how radiation induces AQP4 expression changes, how AQP4 regulates downstream signaling pathways, and how AQP4 changes cell-cell tight junctions in the brain tissue, the optimal time window for AER-271 treatment and how it can be transferred to clinical applications, and whether AQP4 treatment can improve advanced cognitive impairment. More research is needed to explore these in the future.

## Data Availability

The raw data supporting the conclusion of this article will be made available by the authors, without undue reservation.
